# Morphine versus Hydromorphone: Does Choice of Opioid Influence Outcomes?

**DOI:** 10.1155/2015/482081

**Published:** 2015-11-01

**Authors:** Padma Gulur, Katharine Koury, Paul Arnstein, Hang Lee, Patricia McCarthy, Christopher Coley, Elizabeth Mort

**Affiliations:** Massachusetts General Hospital, Boston, MA, USA

## Abstract

Morphine has traditionally been considered the first line agent for analgesia in hospitals; however, in the last few years there has been a shift towards the use of hydromorphone as a first line agent. We conducted a hospital population based observational study to evaluate the increasing use of hydromorphone over morphine in both medical and surgical populations. Additionally, we assessed the effect of this trend on three key outcomes, including adverse events, length of stay, and readmission rates. We evaluated data from the University Health Systems Consortium. Data from 38 hospitals from October 2010 to September 2013 was analyzed for patients treated with either hydromorphone or morphine. The use of morphine steadily decreased while use of hydromorphone increased in both medical and surgical groups. Rescue drugs were used more frequently in patients treated with hydromorphone in comparison to patients treated with morphine (*p* < 0.01). Patients receiving morphine tended to stay in the hospital for almost one day longer than patients receiving hydromorphone. However, 30-day all cause readmission rates were significantly higher in patients treated with hydromorphone (*p* < 0.01). Our study highlights that the choice of hydromorphone versus morphine may influence outcomes. There are implications related to resource utilization and these outcomes.

## 1. Introduction

In 2001, it was reported that approximately 9 in 10 Americans frequently experience pain [1]. In the United States, it has been estimated that 100 million adults suffer from chronic pain alone [2]. As a result, arguments for the undertreatment of pain in the United States have led to several outcomes. The American Pain Society (APS) released guidelines in 1995, which stated that the first step towards improving pain management is assessment and recording of patients' pain reports [3]. The Joint Commission addressed the common practice of suboptimal pain management in 2001 by requiring professionals to ask patients about their pain, treat it when necessary, and evaluate the effects of therapy rendered [4]. At about the same time, the Veterans Administration rolled out a Quality Improvement initiative calling for pain the be treated as the fifth vital sign [5]. Both of these initiatives, confronting the undertreatment of pain, contributed to practice changes that increased awareness on the importance of treating pain. These changes are notable considering that Fox and colleagues stated that the most common symptom that motivates people to seek health care is pain [6]. In turn, there have been unprecedented increases in opioid production and prescription in attempt to manage pain in the United States [5, 7]. Specifically, 80% of the worldwide opioid supply is consumed by Americans, who make up less than 5% of the world's total population [8].

An interesting national trend appears to be the increasing use of hydromorphone compared to morphine. Some evidence in the literature has supported this change [9–11]. This is supported by the US aggregate production data of opioids, which shows a 261.52% increase in hydromorphone production compared to a 190.19% increase in morphine production from 2003 to 2013 [12]. The increase in production and therapeutic use of opioids has paralleled an alarming increase in the diversion, misuse, and development of addiction disorders to prescription opioids. Hydromorphone use, in particular, may contribute to these threats to public health given a faster onset of action and greater euphoric effects when compared to morphine [13].

Morphine is a benzylisoquinoline alkaloid which acts as a potent *μ*-opioid receptor agonist [14–16]. Morphine is metabolized by glucuronidation into morphine-3-glucuronide (M3G) and morphine-6-glucuronide (M6G) which are active metabolites.

Hydromorphone is a semisynthetic opioid agonist which also acts as a potent *μ*-opioid receptor agonist and is synthesized somewhat easily by modifying morphine [14]. In comparison to morphine, hydromorphone has a shorter half-life and a greater impact on sedation [13]. Hydromorphone does not form an active 6-glucuronide metabolite; however, it does have the 3-glucoronide metabolite (H3G) which has been shown to have neurotoxic side effects [17, 18].

Studies have shown that morphine and hydromorphone at equianalgesic doses are very similar and there is no difference in side effect profile [10, 15, 19]. The street value of hydromorphone is higher than morphine, which is an indicator of its likability and recreational use potential [13]. Clinical practice drives the prescription drug abuse epidemic. Hence there is increasing national and international concern regarding the use of hydromorphone [20–23].

The purpose of our study was to evaluate the increasing use of hydromorphone over morphine for analgesia in hospitals. Further, we looked at the impact of this trend on outcome measures such as opioid related adverse events, length of stay, and readmission rates.

## 2. Methods

This hospital population based observational study evaluated data collected from the University Health Systems Consortium (UHC) Clinical Data Base/Resource Manager (CDB/RM). This is an alliance of 118 academic medical centers and 298 of their affiliated hospitals. It accounts for more than 90% of the not for profit academic medical centers in the United States. The UHC CDB/RM is a comparative database with patient level all-payer hospital UB-92 and discharge abstract data from these academic centers and their affiliates. Approval for the use of UHC hospital level data was waived from the Institutional Review Board at Massachusetts General Hospital as this data analysis did not meet the definition of human subjects' research.

The study period was from October 2010 to September 2013. Hospitals with over 500 beds that had reported data for all of our fields of interest during the study period were included. UHC currently has 74 hospitals with over 500 beds in the CDB/RM. Hospitals with incomplete data were excluded. 38 hospitals met all the criteria for inclusion. We collected this data using the UHC CDB/RM 2012 Risk Adjustment Model.

We queried the database for patients, of all ages, who had received either morphine or hydromorphone during their hospital stays but not both drugs (single treatment). If a patient was hospitalized more than once and received either hydromorphone or morphine, then that patient was included again in this data. The APR-DRG (all patient refined-diagnosis related groups) severity of illness (SOI) was collected for our cohorts to help us compare the groups.

We collected outcome data on length of stay, which does not include emergency department time. Additionally, we gathered data on 30-day all cause readmission rates and adverse events. For the purposes of this study, adverse events were defined as the use of a rescue drug (naloxone) during an episode of care. Specifically, naloxone administration on the same day, at any time after the patient received an opioid, was considered an adverse event.

Statistical comparisons of the rates of rescue drug use and 30-day readmission were performed by Chi-square test and 95% confidence intervals, which were constructed by using normal approximation. The 95% confidence intervals of mean lengths of stay were obtained by using normal approximation, and the test was made by independent samples *t*-test.

## 3. Results

### 3.1. Demographics

Averages for demographics were calculated across all three years for patients treated with morphine or hydromorphone. The average age for patients treated with morphine was 49.5 years in comparison to 52.5 years for patients treated with hydromorphone (*p* = 0.995). Of patients treated with hydromorphone, 52.8% were female and 47.2% were male in comparison to patients treated with morphine, 55.4% of which were female and 44.6% were male. Additionally, race was calculated for this population and the majority of patients treated with hydromorphone or morphine were white, 70.8% and 59.8% of each group, respectively.

### 3.2. Medical and Surgical Groups

Over the three years, 628,910 patients were treated with hydromorphone and 751,692 patients were treated with morphine. These patients were further grouped by medical or surgical admissions and this formed our cohort. In the medical cohort, 217,521 patients were treated with hydromorphone and 395,354 were treated with morphine. In the surgical cohort, 411,389 patients were treated with hydromorphone and 356,338 patients were treated with morphine.

### 3.3. Use of Hydromorphone and Morphine

**Figure 1:**
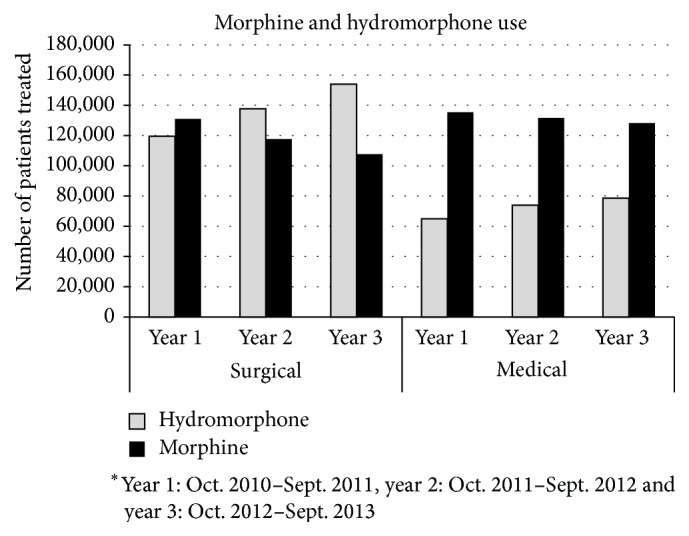
Morphine and hydromorphone use.

Over the three-year study period, the use of morphine steadily decreased while use of hydromorphone increased in both medical and surgical groups. Specifically, we found that hydromorphone use increased by 22% in surgical patients and by 17% in medical patients. On the other hand, morphine use decreased by 22% in surgical patients and by 6% in medical patients. A noteworthy change occurred over the study period as hydromorphone overtook morphine as the more commonly used analgesic in surgical patients (see Figure 1).

### 3.4. Severity of Illness (SOI) of the Cohort

APR-DRG SOI is used to evaluate patient complexity and hospital resource use. SOI is classified into minor, moderate, major, and extreme.

We compared the hydromorphone and morphine groups within the medical and surgical cohorts for their APR-DRG SOI compositions. In the medical cohort, for patients treated with morphine, 49.7% of patients were in the mild and moderate classes and 50.3% of patients were in the major and extreme classes; in turn, for patients treated with hydromorphone, 50.69% patients were in the mild and moderate classes and 49.31% of patients were in the major and extreme classes (*p* < 0.01). In the surgical cohort, for patients treated with morphine, 75.37% of patients were in the mild and moderate classes and 24.62% of patients were in the major and extreme classes; in turn, for patients treated with hydromorphone, 74.04% of patients were in the mild and moderate classes and 25.96% of patients were in the major and extreme classes (*p* < 0.01) (see Table 1).

### 3.5. Adverse Events/Rescue Drug Use

Our results show that rescue drugs are used more often in patients treated with hydromorphone than patients treated with morphine. In patients treated with hydromorphone, rescue drug use was 0.25% higher in the medical group and 0.63% higher in the surgical group (*p* < 0.01) (see Table 2).

### 3.6. Length of Stay

The data on length of stay (LOS), which was measured in days, showed that patients receiving morphine were in the hospital longer than patients receiving hydromorphone. In patients treated with morphine, we found that the average LOS was 0.88 days longer in the medical group and 0.62 days longer in the surgical group (*p* < 0.01) (see Table 2).

### 3.7. 30-Day Readmission

The data shows that the 30-day readmission rate was greater amongst patients receiving hydromorphone when compared to patients receiving morphine. In patients treated with hydromorphone, the all cause 30-day readmission rate was 1.37% higher in the surgical group and 3.41% higher in the medical group (*p* < 0.01) (see Table 2).

## 4. Discussion

In comparing our hydromorphone and morphine groups by APR-DRG SOI, we found a slightly higher percentage of sicker patients in the morphine group compared to the hydromorphone group in both the medical and surgical cohorts (*p* < 0.01). This was statistically different; however, the clinical relevance of a 1% difference in this large population based observational study is not immediately apparent.

The results show that rescue drug use is higher in patients treated with hydromorphone compared to morphine. The main pharmacodynamic difference between hydromorphone and morphine is potency, such that hydromorphone is five to ten times more potent than morphine [10, 15, 24]. Hydromorphone crosses the blood brain barrier faster, resulting in quicker onset and peak of analgesic activity. A study of nonsurgical patients admitted to 288 hospitals in the United States showed that patients prescribed hydromorphone received nearly triple the strength of opioid when compared to patients prescribed morphine [25]. This may help explain the greater use of naloxone among hydromorphone patients found in this study [13]. Adverse events, as defined in our study by rescue drug use, have been directly implicated in increasing healthcare costs [26].

Patients treated with morphine remained in the hospital for a little under one day longer when compared to patients treated with hydromorphone. However, 30-day all cause readmission rates were significantly higher in patients treated with hydromorphone in both the medical and surgical cohorts (*p* < 0.01). Historically, shorter LOS has been equated with lower costs [27]. However, higher readmission rates, which would imply premature discharges, have significant reimbursement implications. In the new hospital value-based purchasing program, reimbursement rates to institutions can be adversely affected by costs incurred when patients are readmitted to the hospital within 30 days after discharge. This could translate to a 2% reduction in total Medicare payments to a hospital in 2014 and a 3% reduction in 2015 [28].

Limitations of our study include the observational nature of the study and the lack of risk adjustments between the groups. Of interest, however, in both medical and surgical study groups, there were more patients treated with morphine alone who were admitted in the severe and extreme severity of illness categories than those treated with hydromorphone. While this may explain that patients treated with morphine stayed in the hospital longer, the higher rescue drug use and readmission rates in patients treated with hydromorphone alone are not in keeping with this observation. In addition, there are limitations with using 30-day all cause readmission rates because unrelated diseases or acute conditions that may be the cause of an additional hospitalization may be lumped together. Lastly, UHC uses billing data, which has inherent limitations as well. For example, we are unable to differentiate patients that received patient-controlled analgesia in comparison to an intravenous bolus.

The increasing use of hydromorphone over morphine does not appear to be supported by recent literature. Our study highlights that the choice of hydromorphone versus morphine may well influence outcomes such as rescue drug use/rate of adverse events, length of stay, and readmission rates. Further research in risk adjusted models may help further delineate these observations. Specifically, data on the underlying diseases of the patient populations could help provide insight into the relative risk for these patients.

## Figures and Tables

**Table 1:** Severity of Illness.

Drug	Medical		Surgical	
	Minor/moderate	Major/extreme	Minor/moderate	Major/extreme
Hydromorphone	50.69%	49.31%	75.37%	24.62%
Morphine	49.70%	50.30%	74.04%	25.96%
*p* value	<0.01	<0.01	<0.01	<0.01

**Table 2:** Outcomes by medical surgical and hydromorphone and morphine.

Outcome	30-day readmission rate		Length of stay		Rescue drug use	
Group	Surgical [95% CI]	Medical [95% CI]	Surgical [95% CI]	Medical [95% CI]	Surgical [95% CI]	Medical [95% CI]
Hydromorphone	4.88% [4.82%–4.95%]	10.15% [10.02%–10.27%]	6.21 [6.20–6.23]	5.68 [5.66–5.69]	2.19% [2.14%–2.23%]	1.11% [1.07%–1.16%]
Morphine	3.51% [3.45%–3.57%]	6.54% [6.46%–6.62%]	6.83 [6.81–6.85]	6.56 [6.54–6.58]	1.56% [1.52%–1.60%]	0.86% [0.83%–0.89%]
*p* value	<0.01	<0.01	<0.01	<0.01	<0.01	<0.01
